# Maternal Dietary Patterns and Pregnancy Outcome

**DOI:** 10.3390/nu8060351

**Published:** 2016-06-07

**Authors:** Xuyang Chen, Diqi Zhao, Xun Mao, Yinyin Xia, Philip N. Baker, Hua Zhang

**Affiliations:** 1 Department of Obstetrics and Gynaecology, The First Affiliated Hospital of Chongqing Medical University, No. 1 Youyi Road, Yuzhong District, Chongqing 400016, China; cxy522454363@163.com (X.C.); 13637790687@163.com (D.Z.); mx1653598066@163.com (X.M.); 2 Canada-China-New Zealand Joint Laboratory of Maternal and Fetal Medicine, Chongqing Medical University, Chongqing 400016, China; kendraxia@163.com (Y.X.); philip.baker@le.ac.uk (P.N.B.); 3 School of Public Health and Management, Chongqing Medical University, Chongqing 400016, China; 4 College of Medicine, Biological Sciences and Psychology, University of Leicester, P.O. Box 138, Leicester LE1 9HN, UK

**Keywords:** dietary patterns, diet, pregnancy, maternal outcome, offspring outcome

## Abstract

Maternal nutritional status during pregnancy will affect the outcomes for the mother and the baby. Many analyses of the relationship between diet and outcome are often based on a single or a few food items or nutrients. However, foods are not consumed in isolation and dietary patterns can be used to assess the whole diet consumed. The use of dietary pattern analysis to understand nutritional intake and pregnancy outcome is becoming more and more popular. Many published studies have showed the association between maternal dietary patterns and pregnancy outcome. This review examined articles about the relationship between maternal dietary patterns and pregnancy outcome. As a modifiable factor, dietary patterns may be more applicable to clinical and pregnant health interventions.

## 1. Introduction

Nutritional status during pregnancy will affect the outcomes for the mother and the baby, but the evidence for a beneficial effect of specific nutritional supplements for women of reproductive age is inconclusive [1,2,3]. Thus, identifying what foods should be eaten, in what quantities, and even how often these foods should be eaten, remain important questions—the answers will enable accurate nutritional assessment and facilitate appropriate counseling in pregnancy.

Many analyses of the relationship between diet and an outcome are based on a single or a few food items or nutrients. Examples such as a high consumption of saturated fatty acids [4,5], total carbohydrates and soft drinks [6,7,8] and a low intake of polyunsaturated fatty acids [9,10] have been associated with increased risk of gestational diabetes mellitus (GDM). The calcium supplementation has been found to decrease preeclampsia [11] and result in fewer preterm births [12]. However, foods are not consumed in isolation and people eat foods containing a mix of nutrients and nonnutrients. Moreover, the individual nutrient approach does not take biological complexity resulting from interactions between nutrients into consideration. For this reason, methods that identify patterns of food intake instead of looking at single food have become popular and are being used to examine potential disease risk prediction [13].

Dietary patterns study the whole diet and provide a simple but helpful, comprehensive and complimentary approach way to deliver meaningful results to the respective population [14]. Dietary patterns have potential to be used as a valid tool in assessing the relationship between diet and pregnancy outcomes [15].

## 2. Methods

The purpose of this review is to examine articles about maternal dietary patterns before or during their pregnancies and pregnancy outcome. For this purpose, a PubMed Central search was conducted up to November 2015. The terms used to search abstracts were (dietary pattern or diet or dietary) AND (pregnancy or pregnant women or infant or offspring). This review included studies of dietary patterns and pregnancy outcome meeting the following criteria: studies based on dietary patterns rather than any single food and studies relating diet during pregnancy with health outcomes in the mother and the infant. The PubMed Central search strategy yielded a total amount of 2927 articles. After applying the inclusion criteria by screening titles and abstracts, 54 articles were selected.

## 3. Dietary Patterns Assessment

The information obtained from food frequency questionnaires (FFQs) can be used to assess dietary patterns. Use of the FFQs has shown the validity of identifying maternal dietary patterns and of studying the relationship between dietary patterns [14]. FFQs have become the most common measure of diet to obtain dietary patterns.

Two different approaches for observing overall dietary exposure were evident in the published literature. One approach is based on the popular hypotheses and guidance about the role of nutrients in disease prevention, and the diet is evaluated in accordance with this guidance (Table 1). In this approach, the diet assessed for certain foods or nutrient characteristics, and then the score resulting from the particular dietary exposure is the variable. 

The alternative approach is data driven, using statistical analyses that seek to minimize bias (Table 2). In epidemiologic studies, statistical analyses play a central role in nutritional epidemiology and are used to promote objectivity in the conclusions drawn [16]. Statistical methods like factor analysis or cluster analysis are common *a posteriori* approaches to the identification of dietary patterns [13].

Factor analysis can be considered a pattern detection method that reduces the number of dietary variables by finding correlated dietary variables. Most published studies that have used factor analysis to describe dietary patterns have used principal components analysis (PCA). The PCA is used to reduce a large amount of detailed information into a smaller set of interpretable factors that have the characteristic of explaining the largest amount of variability in the specific patterns [16].

Cluster analysis is a subject-oriented method with the objective of joining together variables placed into different nonoverlapping groups on the basis of some shared characteristics. Members of a cluster tend to be similar but distinct members of another cluster [17].

Another analytical approach in reflecting complex relationships between diet and disease is latent class analysis (LCA). LCA is useful to study unobserved heterogeneity characterized by several unidentified groups that behave differently. Thus group membership is an unobserved random variable and individuals have a predicted probability for belonging to each class, which reflects the uncertainty of class membership [18]. However, it has rarely been used due to its complexity, making it difficult to interpret and apply the results.

In addition, there is clear evidence of social confounders associated with the dietary patterns. Social factors, such as educational levels, financial difficulties and age, need to be accounted for in studies using dietary patterns [19].

## 4. Results

### 4.1. Infertility

Infertility is defined as the failure to achieve a clinical pregnancy after 12 months or more of regular unprotected sexual intercourse [73]. Its prevalence rates range from 6.9% to 9.3% in developing nations and from 3.5% to 16.7% in developed nations [74].

From the Nurses’ Health Study II a prospective cohort study, a diet score was calculated based on variables previously found to predict ovulatory disorder infertility. This ‘fertility diet’ was found to have favorable effects on fertility and was associated with a 69% lower risk of ovulatory disorder infertility (95% CI 29%–86%) [20]. In a Dutch cohort of couples seeking to conceive by means of vitro fertilization or intracytoplasmic sperm injection, researchers found that a preconception Mediterranean diet contributed to successful pregnancy with a 40% increased probability (OR 1.4, 95% CI 1.0–1.9) [37]. Similar results were obtained in a Spain nested case-control study; a greater adherence to the Mediterranean-type dietary pattern enhanced fertility (OR 0.56, 95% CI 0.35–0.95) [38].

### 4.2. Gestational Diabetes Mellitus

Gestational diabetes mellitus is characterized as glucose intolerance is first diagnosed during pregnancy, is one of the most common pregnancy complications, and has serious, long-term consequences for both mother and baby [75].

A prospective study included 13,110 American women who were free of cancer, cardiovascular disease, type 2 diabetes and history of GDM and reported at least one singleton pregnancy between 1992 and 1998 in the Nurses’ Health Study II. This study found that the Western dietary pattern high in red and processed meat was positively associated with the risk of GDM (OR 1.63, 95% CI 1.20–2.21), however, a prudent dietary pattern high in fruit, green leafy vegetables and fish was negatively associated [39]. Another prospective study included 21,411 singleton pregnancies in the Nurses’ Health Study II between 1991 and 2001 and reported that a pre-pregnancy low-carbohydrate dietary pattern with high protein and fat from animal food sources was positively associated with the risk of GDM (OR 1.36, 95% CI 1.13–1.64), whereas association for a pre-pregnancy low-carbohydrate dietary pattern with high protein and fat from vegetable food sources was not found [21].

In a study in 10 Mediterranean countries, the GDM was interpreted both by the American Diabetes Association (ADA) 2010 and the more stringent International Association of the Diabetes and Pregnancy Study Groups (IADPSG) 2012 criteria which require only one abnormal glucose value, instead of two, for the diagnosis. Adherence to a Mediterranean dietary pattern was associated with better glucose tolerance and a lower incidence of GDM (8.0% *vs.* 12.3%, OR = 0.618, by ADA_2010 and 24.3% *vs.* 32.8%, OR = 0.655, by IADPSG_2012 criteria) [22]. Another study concluded that a prudent dietary pattern in pregnancy was associated with decreased risk of GDM (OR 0.54, 95% CI 0.30–0.98), especially among women who were either overweight or obese pre-pregnancy (OR 0.31, 95% CI 0.13–0.75) [40]. In China, He *et al.* found that the vegetable dietary pattern was significantly associated with a lower risk of GDM (RR 0.79, 95% CI 0.64–0.97), while the sweets and seafood pattern was associated with increased risk of GDM (RR 1.23, 95% CI 1.02–1.49) [41]. In an Australian population-based prospective cohort study, a Mediterranean-style dietary pattern pre-pregnancy was inversely associated with GDM risk (RR 0.85, 95% CI 0.76–0.98), moreover, a dietary pattern of meats, snacks and sweets was associated with higher GDM risk (RR 1.38, 95% CI 1.02–1.86) [42].

A randomized clinical trial aimed at identifying the effects of the Dietary Approaches to Stop Hypertension (DASH) diet (rich in fruits, vegetables, whole grains and low-fat dairy products, and contained lower amounts of saturated fats, cholesterol and refined grains with a total of 2400 mg Na/day) on glucose tolerance and lipid profiles in GDM found that in pregnant women with GDM, the DASH eating pattern for four weeks resulted in beneficial effects on glucose tolerance and lipid profiles compared with a control diet [23]. The same authors demonstrated that consumption of the DASH diet by pregnant women with GDM had beneficial effects on fasting plasma glucose, serum insulin levels, Homeostasis Model of Assessment-Insulin Resistance score, plasma total antioxidant capacity, and total glutathione levels [24]. 

The results from the Nurses’ Health Study II indicated that healthy dietary patterns including the alternate Mediterranean Diet (aMED), DASH, and alternate Healthy Eating Index (aHEI) were significantly associated with a lower risk of GDM (aMED:RR 0.76, 95% CI 0.60–0.95; DASH:RR 0.66, 95% CI 0.53–0.82; aHEI:RR 0.54, 95% CI 0.43–0.68) [25]. Moreover, they observed increased adherence to the aMED, DASH, and aHEI dietary patterns with a lower risk of type 2 diabetes mellitus (T2DM) in this large prospective study of women with a history of GDM (aMED:RR 0.60, 95% CI 0.44–0.82; DASH:RR 0.54, 95% CI 0.39–0.73; aHEI:RR 0.43, 95% CI 0.31–0.59) [26]. All three dietary patterns include a high intake of fruit, vegetables, nuts, legumes, soy, fish, seafood, whole grains and low intake of red and processed meats. aMED includes moderate alcohol and higher ratio of monounsaturated fatty acid to saturated fatty acid (MUFA:SFA). aHEI also includes moderate alcohol, in addition to multivitamin use, higher ratio of polyunsaturated fatty acid to saturated fatty acid (PUFA:SFA) and lower intakes of transfat. DASH assesses high intakes of low-fat dairy, lower sweetened beverages and sodium.

### 4.3. Hypertensive Disorders of Pregnancy

Hypertensive disorders of pregnancy (HDPs), including gestational hypertension and preeclampsia, are obstetric complications [43]. The frequency of preeclampsia ranges between 2% and 7% in healthy nulliparous women. Preeclampsia is associated with adverse health outcomes (maternal mortality, perinatal deaths, preterm birth, and intrauterine growth restriction) for both the mother and child [76].

Research in nulliparous pregnant Norwegian women, found that a dietary pattern characterized by high intake of vegetables, plant foods and vegetable oils resulted in a lower risk of preeclampsia (OR 0.72, 95% CI 0.62–0.85), whereas a dietary pattern characterized by a high consumption of meat, sweet drinks, and snacks increased the risk (OR 1.21, 95% CI 1.03–1.42) [44]. In a prospective cohort study, researchers showed that low adherence to a Mediterranean-style dietary pattern and high adherence to a traditional dietary pattern during pregnancy were positively associated with higher blood pressure during pregnancy, but were not associated with gestational hypertension or preeclampsia outcomes [45]. In a population-based study of Australian women, pre-pregnancy consumption of a Mediterranean-style dietary pattern was associated with lower risk of developing HDPs (RR 0.58, 95% CI 0.42–0.81) [43].

In contrast to the above studies, in a Brazilian cohort, investigators did not observe associations between dietary patterns and prospective changes in blood pressure during pregnancy and the early postpartum period. The difference may be explained on the basis that this study only included healthy pregnant women and excluded those who developed hypertension or preeclampsia. The investigators hypothesized that the powerful mechanisms involved in maternal hemodynamic adaptations, such as the integrity of endothelium, were not disrupted in normotensive pregnant women, and maintained blood pressure despite the different dietary intakes [46].

### 4.4. Depressive Symptoms

The prevalence of depressive symptoms during pregnancy varies from 8% to 26% around the world [77]. Depressive symptoms have been associated with adverse outcomes for both mother and fetus, such as insufficient weight gain, premature birth, low birth weight, obstetric complications, and postpartum depression [78,79,80,81].

From a ‘Rhea’ cohort study in Crete, Greece, intake of the ‘health-conscious’ pattern comprised vegetables, fruit, nuts, pulses, fish and seafood, olive oil and dairy products led to a protective effect against depressive symptoms in postpartum women (RR 0.51, 95% CI 0.25–1.05) [47]. Another study found that traditional (OR 0.84, 95% CI 0.73–0.97) and health-conscious (OR 0.77, 95% CI 0.65–0.93) dietary patterns were associated with protective effects on symptoms of anxiety whereas there was an increased possibility of anxiety symptoms with the vegetarian pattern (OR 1.25, 95% CI 1.08–1.44) [48]. This is due to the reduced animal sources meat and fish in the vegetarian pattern; meat is the richest source of Vitamin B12 and fish is the main source of long-chain essential *n*-3 PUFA, especially docosahexaenoic acid (DHA; 22:6*n*-3). These nutrients are known to be necessary components for optimal neurological function [82]. The direction of causality between observed association between the vegetarian diet and increased maternal anxiety requires further exploration. In a prospective cohort of Brazilian women, researchers found that a pre-pregnancy healthy pattern (the common-Brazilian or healthy patterns) was inversely associated with depressive symptoms throughout pregnancy, even to early postpartum [49,50].

In the Osaka Maternal and Child Health Study, researchers hypothesized that a dietary pattern high in the main food sources of nutrients that are potentially protective against postpartum-depressive symptoms would be related to a lower risk of postpartum depression. However, there was no association between dietary patterns and the risk of depressive symptoms [51].

### 4.5. Preterm Birth

Preterm birth, defined as delivery before 37 weeks or 259 days of gestation occurs in about 10% of pregnancies globally, is significantly associated with neonatal mortality and morbidity, and has long-term adverse consequences for health [83,84]. 

In Denmark, a Mediterranean diet during pregnancy was associated with reduced risk of preterm birth (OR 0.61, 95% CI 0.35–1.05) [27]. Results from a large prospective cohort study in Norway, showed that a “traditional” dietary pattern or a “prudent” dietary pattern during pregnancy was associated with a reduced risk of preterm birth, 9% (OR 0.91, 95% CI 0.83–0.99) and 12% (OR 0.88, 95% CI 0.80–0.97) respectively [52]. In another large, prospective cohort of close to 60,000 Danish women followed during pregnancy, researchers found that a high intake of Western-type diet led to an increased risk of preterm birth (OR 1.30, 95% CI 1.13–1.49), while a seafood diet had a modestly protective effect (OR 0.90, 95% CI 0.72–1.11) [53]. Moreover, a study of the association between preconception dietary patterns and preterm birth demonstrated that a high-protein/fruit pattern reduced the likelihood of induced preterm birth (OR 0.31, 95% CI 0.13–0.72) and a high-fat/sugar/takeaway was positively associated with preterm birth (OR 1.54, 95% CI 1.10–2.15) and shorter gestational ages [54]. In contrast, in other studies there was no association between Mediterranean diet adherence during pregnancy and the risk of preterm birth [28,29], although a mother-child cohort study found that adherence to the Mediterranean diet was associated with a lower risk of preterm birth, specifically in overweight and obese women (OR 0.7, 95% CI 0.6–0.9) [29].

### 4.6. Fetal Growth

The baby’s health outcome is the single issue that a mother cares most about. Fetal growth is an important determinant of future health and development [85,86,87].

A high adherence to a maternal prudent diet (characterized by a high intakes of fruit and vegetables, whole meal bread, rice, and pasta, yogurt, and breakfast cereals and low intakes of chips and roast potatoes, sugar, white bread, processed meat, crisps, tinned vegetables, and soft drinks) was found to be associated with greater offspring bone size and bone mineral density; this study suggested that maternal dietary pattern during pregnancy was an independent determinant of bone mineral accrual in the offspring [55]. Through a prospective study of the Danish National Birth Cohort, researchers indicated that the maternal Western diet was positively associated with offspring forearm fractures (hazard ratio 1.11, 95% CI 1.01–1.23) [56].

Four studies have observed the relationship between maternal dietary patterns during pregnancy and subsequent small for gestational age (SGA) infants. A Danish study has found that the Western diet led to a significantly higher risk of having a SGA infant and lower birth weight [57]. In New Zealand, Thompson *et al.* found that the ‘traditional’ diet in early pregnancy was associated with a lower risk of having a SGA infant (OR 0.86, 95% CI 0.75–0.99) [58]. A Dutch study indicated that high adherence to a Mediterranean dietary pattern was associated with decreased risk of SGA [30]. In Japan, pregnant women in the ‘wheat products’ pattern had significantly higher probabilities of having a SGA infant (multivariate OR 5.2, 95% CI 1.1–24.4) [59].

Studies conducted in UK and Brazil both found a positive association between dietary pattern and birth weight, a ‘health conscious’ pattern in UK and a snack dietary pattern in Brazil [60,61]. Moreover, another study from the Netherlands suggested that high adherence to an energy-rich dietary pattern was associated with increased crown–rump length in the first trimester [62].

In contrast, two studies found that no dietary pattern was significantly associated with any of the birth outcomes [29,63]. An adherence to Mediterranean diet was not significantly associated with the risk of delivering an infant with fetal growth restriction [29]. No dietary pattern was significantly associated with any of the birth outcomes, the mixed dietary patterns may provide antagonistic relationships between foods and nutrients that result in null associations with birth outcomes [63].

### 4.7. Asthma

The prevalence of asthma and allergies in children has been increasing globally during the last few decades [88].

Four studies have examined the relationship between a maternal Mediterranean diet during pregnancy and asthma or other atopic conditions in children. Two studies found a protective effect of a high level of adherence to a Mediterranean diet during pregnancy against childhood atopic conditions at ages 6–7 years [31,32]. However, the other two studies suggested that a Mediterranean diet during pregnancy was not associated with infantile wheeze and eczema [33,34].

A Japanese study examined three maternal dietary patterns (Healthy, Western, and Japanese). The maternal Western pattern during pregnancy may have had a preventive effect against wheeze in offspring (OR 0.59, 95% CI 0.35–0.98), however, neither the Healthy diet nor Japanese diet during pregnancy was related to wheeze or eczema in offspring [64]. Two other studies investigated the association between maternal dietary patterns during pregnancy and wheeze, but none of the maternal dietary patterns was meaningfully associated with wheeze in the offspring [35,65].

A further study investigated the relationship between maternal diet and maternal asthma control during pregnancy. This study found pre-pregnancy dietary patterns influenced maternal asthma control [66].

### 4.8. Others

A small number of studies have investigated the association between dietary patterns and other pregnancy/offspring outcomes.

There are two global domains of child problem behavior: internalizing problems consist of emotionally reactive, anxious/depressed symptoms, somatic complaints and symptoms of being withdrawn; externalizing problems consist of attention problems and aggressive behavior. Both high adherence to the traditionally Dutch diet characterized by a high intakes of fresh and processed meat, potatoes, margarines and low intake of soy and diet products (OR 1.11, 95% CI 1.03–1.21) and low adherence to the Mediterranean diet (OR 0.90, 95% CI 0.83–0.97) during pregnancy are significantly associated with an increased risk of childhood externalizing problems; there were no associations between dietary patterns and child internalizing problems [67].

There are some studies of the congenital defect about babies. In contrast to the prudent diet (characterized by a high intakes of fish, garlic, nuts, and vegetables and a higher frequency of hot meals per day) users, mothers with the high adherence to the Western diet increased the risk of having an offspring with a cleft lip or cleft palate approximately two fold (OR 1.9, 95% CI 1.2–3.1) [68]. The Mediterranean dietary pattern seemed to have a protective effect in reducing the risk of offspring being affected by spine bifida [69]. A more ‘Non-health conscious’ maternal food pattern was associated with an increased risk of hypospadias in baby boys (OR 1.54, 95% CI 1.06–2.26) [70]. Women who adhered to a prudent dietary pattern, even when folate supplementation was administered, may have lower risk of neural tube defects (NTDs) and some heart defects [71].

When cancer in offspring was studies, Musselman *et al.* found that higher adherence to a diet that is high in fruits and vegetables was associated with a lower likelihood of childhood germ cell tumors (OR 0.83, 95% CI 0.69–0.99) [72]. A case-control study provided preliminary evidence that a diet higher in fruit and lower in fried foods and cured meats during pregnancy may decrease the risk of unilateral retinoblastoma in offspring (OR 0.75, 95% CI 0.61–0.92) [36].

## 5. Conclusions

The validity of identifying maternal dietary patterns and of studying the relationship between dietary patterns using FFQs has been demonstrated [14]. The majority of studies in this review used FFQs rather than diet scores to obtain a comprehensive account of dietary information. However, there are inevitable misclassifications in dietary intake; this is likely to bias the magnitude of the observed effects towards the null hypothesis. Moreover, as most of the diet information was obtained from memory, limitations of recall bias are also inevitable.

In a mother–child ‘Rhea’ cohort in Crete, Greece, two dietary patterns during pregnancy were identified using principal component analysis: ‘health conscious’ and ‘Western’. The ‘Western’ dietary pattern comprised mainly meat and meat products, potatoes, sugar and sweets, cereals, fats except olive oil, salty snacks, eggs, beverages and sauces [47]. In contrast, the Western pattern in a Japan study was characterized by low consumption of soft drinks and sweets [64]. The Western pattern in Greece may thus have been more extreme than the Western pattern in Japan. This is just one example of different dietary patterns in the different studies having the same name. In contrast, some patterns that have similar content have an alternative name in different studies. There is a variety of names for the ‘Mediterranean Diet’, such as Mediterranean-type diet, Mediterranean Diet Index, alternate Mediterranean Diet and Mediterranean Diet Score. Confusingly, the main diet content are similar, but the methods for calculation are subtly different, and dietary criteria to define the Mediterranean Diet was used for some studies [27,28] while much research used the method of scoring according to the consumption of various foodstuffs [22,25,26,29,30,31,32,33,34,35]. Therefore, a comparison between various research studies is both difficult and challenging and formal development of taxonomy and classification is required.

This review has highlighted many research studies of dietary patterns associated with pregnancy outcome, however, some pregnancy outcomes have yet to be investigated, such as intrahepatic cholestasis of pregnancy.

In addition, low-glycemic-index (GI) diet has also impact on pregnancy outcomes [89,90,91]. In recent years, low-GI diet has received increased interest from the public and researchers as an important strategy in GDM management [92]. However, low-GI diet mainly focuses on the glycemic index of food and its carbohydrate content, not the overall diet. We did not include it in our review. Also, there is a comprehensive evaluation of GI and pregnancy outcomes [92].

The studies detailed above highlight the importance of emphasising healthy dietary choices in preconception counseling to optimise not only reproductive outcomes but also general maternal health. Current guidelines of preconception care emphasise that nutrition and certain lifestyle factors play an important role in pregnancy. This review finds evidence that, for European countries, the Mediterranean diet is a relatively healthy diet. Importantly, the diets with higher intake of fruits, vegetables, legumes and fish have positive pregnancy outcomes in general and this conclusive evidence should be communicated to women specifically. As a modifiable factor, diet is a key area for intervention in pregnant women, but the precise content of the intervention is yet to be elucidated.

## Figures and Tables

**Table 1:** Studies reporting dietary patterns based on the popular hypotheses and guidance about the role of nutrients in disease prevention.

Reference	Dietary Patterns Assessment	Food	Distinct Nutrients
Chavarro, J.E., *et al.* [20]	FFQ ^a^ (items NA ^b^) “Fertility diet” score	High “fertility diet” score was characterized by a higher intake of monounsaturated fat, vegetable protein, high-fiber, low-glycemic carbohydrates, high fat dairy products, nonheme iron intake and higher frequency of multivitamin use, but a lower intake of trans fat and animal protein.	Multivitamins, vegetable protein and the amount and quality of carbohydrates.
Bao, W., *et al.* [21]	FFQ (items NA) Low-carbohydrate dietary patterns scores	Higher scores consumed more heme iron, red meat, poultry, and high fat dairy but less total calories, dietary fiber, magnesium, vitamin C, vitamin E, low-fat dairy, fruit, vegetables, whole grains, and sugar-sweetened beverages.	Fat, protein, carbohydrate, heme iron and nitrite.
Karamanos, B., *et al.* [22]	78 questions dietary questionnaire Mediterranean Diet Index	Evaluated diet for intake of bread, cereals, legumes, vegetables, fruits, meat, fish, eggs, the ratio of olive oil to animal fat, potatoes, cheese and dairy products.	*trans* fatty acids, fiber, antioxidants, polyphenols and magnesium.
Asemi, Z., *et al.* [23,24]	Dietary Approaches to Stop Hypertension	Rich in fruits, vegetables, whole grains and low-fat dairy products, and contained lower amounts of saturated fats, cholesterol and refined grains with a total of 2400 mg Na/day	Dietary fiber, phytoestrogens, cholesterol, potassium, magnesium, folic acid.
Tobias, D.K., *et al.* [25,26]	FFQ (items NA) Alternate Mediterranean Diet Dietary Approaches to Stop Hypertension Alternate Healthy Eating Index	High intakes of fruit, vegetables, nuts, legumes, soy, fish, seafood, whole grains, moderate alcohol and higher MUFA:SFA ^c^, but low intakes of red and processed meats. High intakes of fruit, vegetables, nuts, legumes, soy, fish, seafood, whole grains and low-fat dairy, but low intakes of red, processed meats, sweetened beverages and sodium. High intakes of fruit, vegetables, nuts, legumes, soy, fish, seafood, cereal fiber, moderate alcohol, multivitamin use and higher white: red meat ratio and PUFA:SFA ^d^, but low intakes of trans fat.	Antioxidants and photochemicals, dietary fiber, saturated fat, heme iron, nitrosamines and micronutrients such as magnesium and vitamin C.
Mikkelsen, T.B., *et al.* [27]	360-items FFQ Mediterranean-type diet	Consumption of fish twice a week or more (lunch or dinner), intake of olive or rape seed oil, high consumption of fruits and vegetables (5 a day or more), meat (other than poultry and fish) at most twice a week, and at most 2 cups of coffee a day.	Higher carbohydrate, vitamin C, folate, α-tocopherol, magnesium, calcium, iron and vitamin D intake and lower sugar, alcohol and cholesterol.
Haugen, M., *et al.* [28]	255-items FFQ Mediterranean-type diet	Intake of at least 5 vegetables/fruits per day, 2 servings of fish or more per week, olive or canola oil for cooking and dressings, no more than 2 servings of red meat per week, and no more than 2 cups of coffee per day.	Higher intake of vitamin C, folate and fiber and lower intake of fat and saturated fat.
Saunders, L., *et al.* [29]	214-items FFQ The Mediterranean scale	Evaluated diet for intake of beneficial components (vegetables, legumes, fruits and nuts, cereals, fish and dairy products), components presumably detrimental (meat, poultry and alcohol) and fat.	Vitamins C, E and *n*-3 PUFA.
Timmermans, S., *et al.* [30]	293-items FFQ Adherence to the Mediterranean diet	Higher intakes of pasta, rice, vegetable oils, fish, vegetables and alcohol, and lower intakes of meat, potatoes and fatty sauces.	Vegetable protein, carbohydrate polymers, fiber, folate, vitamin B12 and a favourable ratio of unsaturated: saturated lipids.
Chatzi, L., *et al.* [31]	42-items FFQ Mediterranean Diet Score	Evaluated diet for intake of vegetables, legumes, fruits and nuts, cereal, fish, dairy products and meat.	Antioxidant compounds (vitamin C, E, phenolic acids, phytic acid, carotenoids, selenium, flavonoids) and *n*-3 PUFA.
de Batlle, J., *et al.* [32]	70-items FFQ Mediterranean diet score	A greater adherence to a Mediterranean dietary pattern is a high intake of vegetables, fruits and nuts, legumes, fish and cereals, and a low intake of dairy products, meat and junk food and fat.	High ω-3/ω-6 fatty acid ratio and low salt content.
Castro-Rodriguez, J.A., *et al.* [33]	The environmental questionnaire included questions on the consumption of certain foods based on the International Study of Asthma and Allergies in Childhood Phase III Mediterranean diet score	Evaluated diet for intake of fruit, fish (oily and white), vegetables (including salads), legumes, cereals, pasta, rice, potatoes, meat, milk and fast food.	Low in SFA and high in carbohydrates, fiber, antioxidants, MUFA and *n*-3 PUFA.
Chatzi, L., *et al.* [34]	100-items FFQ in Spain 250-items FFQ in Greece Mediterranean diet score	Evaluated diet for intake of beneficial components (vegetables, legumes, fruits and nuts, cereals, fish and seafood, and dairy products), components presumably detrimental (meat, including all types of meat) and fat.	Low in SFA and high in carbohydrates, fiber, antioxidants, MUFA and *n*-3 PUFA.
Lange, N.E., *et al.* [35]	166-items FFQ Mediterranean diet score Alternate Healthy Eating Index	Evaluated diet for intake of beneficial components (dairy, fish, fruit, legumes, nuts, vegetables, unsaturated-to-saturated fat ratio, and whole grains) and components presumably detrimental (red and processed meats).	NA
		Evaluated diet for intake of vegetables; fruit; ratio of white to red meat; fiber; transfat; ratio of polyunsaturated to saturated fatty acids; and folate, calcium, and iron from foods.	NA
Lombardi, C., *et al.* [36]	72-items FFQ Two a priori dietary scores	One capturing a diet high in fruits and vegetables and low in red and cured meats and one capturing a diet high in fruits and vegetables and low in fried foods and sweets.	Folate, vitamin B6, α-carotene, lutein/zeaxanthin, acrylamide and nitrates.

^a^ FFQ: food frequency questionnaire; ^b^ NA: not available; ^c^ MUFA: monounsaturated fatty acid; SFA: saturated fatty acid; ^d^ PUFA: polyunsaturated fatty acid.

**Table 2:** Studies reporting dietary patterns derived from statistical analyses.

Reference	Dietary Assessment	Dietary Patterns	Food	Distinct Nutrients
Vujkovic, M., *et al.* [37]	195-items FFQ ^a^	Health conscious–low Processed	High intakes of fruits, vegetables, whole grains, fish, and legumes, but low intakes of mayonnaise, snacks, and meat products.	NA ^c^
	PCA ^b^	Mediterranean	High intakes of vegetable oil, fish, legumes, and vegetables but low intakes of snacks.	Vitamin B6 and fatty acids.
Toledo, E., *et al.* [38]	136-items FFQ	Western-type	High consumption of processed and unprocessed red meat, fast food, whole fat dairy products, commercial bakery, potatoes, eggs, refined grains, sauces, processed meals, and sugar-sweetened soda.	Carbohydrates, protein and trans unsaturated fat.
	PCA	Mediterranean-type	High consumption of vegetables, fish, fruits, poultry, low-fat dairy products, and olive oil.	Iron intake, polyunsaturated fatty acids, B vitamins and folic acid.
Zhang, C., *et al.* [39]	133-items FFQ	Western	High intake of red meat, sweets, processed meat, refined grain products, French fries and pizza.	Saturated fatty acids and cholesterol nitrites and haem iron.
	PCA	Prudent	High intake of fruit, green leafy vegetables, poultry and fish.	NA
Tryggvadottir, E.A., *et al.* [40]	4-day weighed food record PCA	Prudent	Positive for seafood, eggs, vegetables, fruit and berries, vegetable oils, nuts and seeds, pasta, breakfast cereals, and coffee and tea, and negative for soft drinks and French fries.	Polyunsaturated fatty acids.
He, J.R., *et al.* [41]	67-items FFQ	Vegetable pattern	Evaluated intake of root vegetables, beans, mushrooms, melon vegetables, seaweed, other legumes, fruits, leafy and cruciferous vegetables, processed vegetables, nuts, and cooking oil.	Dietary fiber.
	PCA	Protein-rich pattern	Evaluated intake of poultry, red meat, animal organ meat, grains (mainly refined), processed meat, fish, soups, leafy and cruciferous vegetables, and eggs.	NA
		Prudent pattern	Evaluated intake of dairy products, nuts, eggs, fish, soups, fruits, and infrequent intake of processed meat, sugar-sweetened beverages, and processed vegetables.	NA
		Sweets and seafood pattern	Evaluated intake of Cantonese desserts, mollusks and shellfish, and sugar-sweetened beverages and low intakes of grains (mainly refined) and leafy and cruciferous vegetables.	NA
Schoenaker, D.A., *et al.* [42]	101-items FFQ	Meats, snacks and sweets	High consumption of red and processed meat, cakes, sweet biscuits, fruit juice, chocolate and pizza.	Sugar and saturated fat.
	PCA	Mediterranean-style	High factor loadings for vegetables, legumes, nuts, tofu, rice, pasta, rye bread, red wine and fish.	Dietary fiber, magnesium, Vitamin E and other antioxidants.
		Fruit and low-fat dairy	Positively correlated with fruits and low fat dairy including yoghurt, low-fat cheese and skimmed milk.	NA
		Cooked vegetables	High consumption of carrots, peas, cooked potatoes, cauliflower and pumpkin.	NA
Schoenaker, D.A., *et al.* [43]	101-items FFQ	Meat, high-fat, and sugar	High consumption of meat, processed meat, cakes, sweet biscuits, chocolate, meat pies, and pizza.	NA
	PCA	Mediterranean-style	High consumption of vegetables, legumes, nuts, tofu, rice, pasta, rye bread, red wine, and fish.	Vitamin E, dietary fiber, magnesium, and PUFA ^d^.
		Fruit and low-fat dairy	High consumption of fruit, yogurt, low-fat cheese, and skim milk.	NA
		Cooked vegetable	High consumption of carrots, peas, cooked potatoes, cauliflower, and pumpkin.	NA
Brantsaeter, A.L., *et al.* [44]	255-items FFQ	Vegetable	High positive loadings on vegetables, cooking oil, olive oil, fruits and berries, rice, and chicken.	Folate, Vitamin B6, fiber, Vitamin C, β-carotene, tocopherol, potassium, and magnesium.
	PCA	Processed food	High positive loadings on processed meat products, white bread, French fries, salty snacks, and sugar-sweetened drinks and high negative loadings on oily fish, high-fiber breakfast cereals, and lean fish.	SFA ^e^, added sugar and sodium.
		Potato and fish	High positive loadings on cooked potatoes, processed fish, lean fish, fish spread and shellfish, and margarine.	Sodium, fiber, folate, and tocopherol.
		Cakes and sweets	High loadings on cakes, waffles and pancakes, buns, ice cream, sweet biscuits, sweets, and chocolate.	SFA and added sugar.
Timmermans, S., *et al.* [45]	293-items FFQ	Mediterranean	High intake of vegetables, vegetable oils, pasta, rice, fish, and legumes, moderate intake of alcohol, and low intake of sweets.	Vegetable protein, carbohydrates polymers, fiber, folate, Vitamin B1 and a favorable ratio of unsaturated to saturated lipids.
	PCA	Traditional	High intake of meat and potatoes, and low intake of fruit, nonalcoholic drinks, fish, and bread.	Animal protein and saturated lipids.
Eshriqui, I., *et al.* [46]	82-items FFQ	Healthy	High intake of milk, dairy products, fruit, fruit juice, green vegetables and legumes, fish, cakes and cookies-crackers and tea.	Fatty acids, fiber, magnesium, potassium, calcium, flavonoids and antioxidants.
	PCA	Common-Brazilian	High intake of rice, beans, vegetable spices, eggs and bread and fats.	NA
		Processed	High intake of meats, candies And sugar, pasta, roots and tubers, fast food and snacks, sausages and deli meats, soft drinks, and was inversely related to beef and coffee.	NA
Chatzi, L., *et al.* [47]	250-items FFQ	Western	Mainly comprised meat and meat products, potatoes, sugar and sweets, cereals, fats except olive oil, salty snacks, eggs, beverages and sauces.	NA
	PCA	Health conscious	Mainly comprised vegetables, fruit, nuts, pulses, fish and seafood, olive oil and dairy products.	Vitamins C, E, carotenoids, Se Flavonoids, α- tocopherol, PUFA and polyphenols.
Vaz Jdos, S., *et al.* [48]	43-items FFQ	Health conscious	High intake of salad, fruit, fruit juice, rice, pasta, oat/bran based breakfast cereal, fish, pulses, cheese, non-white bread.	Vitamin B12 and *n*-3 PUFA.
		Traditional	High intake of vegetables, red meat, poultry.	NA
	PCA	Processed	High intake of meat pies, sausages, burgers, fried foods, pizza, chips, white bread, eggs, baked beans.	NA
		Confectionery	High intake of chocolate, sweets, biscuits, cakes, puddings.	NA
		Vegetarian	High intake of meat substitutes, pulses, nuts, herbal tea and high negative loadings for red meat and poultry.	NA
Vilela, A.A., *et al.* [49,50]	82-items FFQ	Common-Brazilian	Consisted of rice, beans, vegetable spices, and meats and eggs.	*n*-3 PUFA.
	PCA	Healthy	Comprised dairy products, fruits and fruit juices, green vegetables and legumes, candies, fish, cakes and cookies/crackers, noodles, pasta, roots and tubers, and tea.	Flavonoids, vitamins, minerals and *n*-3 PUFA.
		Processed	High intake of bread, fat, fast food and snacks, sugar, sausages and deli meats, soft drinks, and coffee.	Fat and carbohydrates.
Okubo, H., *et al.* [51]	150-items FFQ	Healthy	High intake of green and yellow vegetables, seaweeds, white vegetables, potatoes, fish, fruits, shellfish and sea products.	Vitamins B2, B6, B12, folate, total *n*-3 PUFA, DHA and EPA ^f^.
	Factor analysis	Western	High intake of vegetable oil, beef and pork, salt-containing seasonings, processed meat, chicken and eggs.	Total *n*-3 PUFA.
		Japanese	High intake of rice, miso soup, sea products, fish and pickled vegetables.	Total *n*-3 PUFA, DHA and EPA.
Englund-Ogge, L., *et al.* [52]	255-items FFQ	Prudent	High intake of raw and cooked vegetables, salad, onion/leek/garlic, fruit and berries, nuts, vegetables oils, water as beverage, whole grain cereals, poultry, and fiber rich bread, low intake of processed meat products (hot dogs, hamburgers, and so on), white bread, and pizza/tacos.	Folic acid, dietary fibre, β carotene, potassium, and ascorbic acid.
	PCA	Western	High intake of salty snacks, chocolate and sweets, cakes, French fries, white bread, ketchup, sugar sweetened drinks, processed meat products, and pasta, low intake of lean fish and fiber rich bread.	Total fat, saturated fat, and added sugar.
		Traditional	High intake of boiled potatoes, fish products, gravy, lean fish, margarine, rice pudding, low fat milk, an cooked vegetables, low intake of poultry and pizza/tacos.	Potassium, magnesium, protein and dietary fiber.
Rasmussen, M.A., *et al.* [53]	360-items FFQ	Vegetables/Prudent	High in cabbage, onion, mushroom, corn, salad, tomato, vegetables other and legumes	NA
		Western	High in potatoes, french fries, bread white, pork, beef veal, meat mixed, meat cold and dressing sauce.	NA
		Nordic	High in bread dark, fruit nordic, banana, fruit dried, sweet spread and hard cheese.	NA
	PCA	Seafood	High in fish lean, fish oily, fish cold, lamb, egg and vegetables other.	*n*-3 fatty acids.
		Alcohol	High in wine, beer, liquor, soya and root.	NA
		Candy	High in bread white, french fries, margarine, sweet spread, candy, sugar cakes, dessert dairy and chocolate.	NA
		Rice/Pasta/Poultry	High in pasta, rice and poultry	NA
Grieger, J.A., *et al.* [54]	100-items FFQ	High-protein/fruit	High in fish, meat, chicken, fruit, and some whole grains.	Long-chain ω-3 PUFAs, protein, niacin equivalents, cholesterol, zinc, iron, and sodium.
	PCA	High-fat/sugar/takeaway	High in takeaway foods, potato chips, refined grains.	Saturated fat, α-linolenic acid, total fat, energy, carbohydrate and MUFAs.
		Vegetarian-type	High in vegetables, legumes, whole grains.	Fiber, dietary folate and Vitamin A.
Cole, Z.A., *et al.* [55]	100-items FFQ PCA	Prudent diet	High intakes of fruit and vegetables, whole meal bread, rice, and pasta, yogurt, and breakfast cereals and low intakes of chips and roast potatoes, sugar, white bread, processed meat, crisps, tinned vegetables, and soft drinks.	Protein and calcium.
Petersen, S.B., *et al.* [56]	360-items FFQ	Prudent	High in vegetables, legumes, root, fruit, corn and low in meat, french fries, margarine, white bread, candy/snack.	NA
		Alcohol	High in alcohol, soy, root, soft drinks, berries and low in pasta/rice, yogurt, poultry, cheese, bread.	NA
	PCA	Western	High in meat, potatoes, white bread, egg, margarine and low in vegetables, fruit, breakfast, cereals, nuts, water.	NA
		Nordic	High in dark bread, nordic fruit, cheese, banana, cakes and low in french fries, candy/snack, soft drinks, processed meat, desserts.	NA
		Shellfish	High in fish, shellfish, lamb, oils, egg and low in soft drinks, diet, candy/snack, low-fat milk, coffee, white bread.	NA
		Sweets	High in white bread, cakes, margarine, french fries, soft drinks, sugar and low in Low-fat milk, cabbage, fruits, fish, legumes.	NA
		Traditional	High in poultry, meat, low-fat milk, meat, water and low in full fat milk, coffee, butter, potatoes, white bread.	NA
Knudsen, V.K., *et al.* [57]	360-items FFQ	Western	High intake of high-fat dairy, refined grains, processed and red meat, animal fat (butter and lard), potatoes, sweets, beer, coffee and high-energy drinks.	Saturated fats and trans fatty acids.
	PCA	Health conscious	High intake of fruits, vegetables, fish, poultry, breakfast cereals, vegetable juice and water.	Vitamins and minerals.
		Intermediate	Low-fat dairy and fruit Juice, consumption of the remaining food groups was in between western and health conscious.	NA
Thompson, J.M., *et al.* [58]	71-items FFQ	Junk	High intake of ice cream, sweet biscuits, scones, cakes, sweetened cereal, crisps, pies, lollies, chocolate bars, ice blocks and milo (chocolate energy drink).	NA
	PCA	Traditional	High intake of apples/pears, citrus fruit, kiwifruit/feijoas, bananas, green vegetables, root vegetables, peas/maize, dairy food/yogurt and water.	NA
		Fusion	High intake of fruits, fried rice/noodles, boiled rice/pasta, fish/shellfish, milk and negative loading for tea/coffee, sherry/wine and hard cheeses.	NA
Okubo, H., *et al.* [59]	150-items FFQ	Meat and eggs	High intake of beef and pork, processed meat, chicken, eggs, butter and dairy products.	NA
	*K*-means cluster analysis	Wheat products	High intake of bread, confectioneries, fruit and vegetable juice, and soft drinks.	NA
		Rice, fish and vegetables	High intake of rice, potatoes, nuts, pulses, fruits, green and yellow vegetables, white vegetables, mushrooms, seaweeds, Japanese and Chinese tea, fish, shellfish, sea products, miso soup and salt-containing seasoning.	NA
Northstone, K., *et al.* [60]	44-items FFQ	Health conscious	High intake of salad, fruit, rice, pasta, breakfast cereals, fish, eggs, pulses, fruit juices, poultry and non-white bread.	NA
		Traditional	High intake of green vegetables and root vegetables, potatoes, peas and to some extent red meat and poultry.	NA
	PCA	Processed	High intake of high-fat processed foods, such as meat pies, sausages and burgers, fried foods, pizza, chips and crisps.	NA
		Confectionery	High intake of confectionery and other foods with high sugar content such as chocolate, sweets, biscuits, cakes and other puddings.	NA
		Vegetarian	High intake of meat substitutes, pulses, nuts and herbal tea and low intake of red meat and poultry.	NA
Coelho Nde, L., *et al.* [61]	29-items FFQ	Prudent pattern	High intake of milk, yogurt, cheese, fruit and fresh-fruit juice, cracker, and chicken/beef/fish/liver.	NA
	PCA	Traditional	High intake of beans, rice, vegetables, breads, butter/margarine and sugar.	NA
		Western	High intake of potato/cassava/yams, macaroni, flour/farofa/grits, pizza/hamburger/deep fried pastries, soft drinks/cool drinks and pork/sausages/egg.	NA
		Snack pattern	High intake of sandwich cookie, salty snacks, chocolate, and chocolate drink mix.	High simple carbohydrates, lipids and low amounts of protein and micronutrients.
Bouwland-Both, M.I., *et al.* [62]	293-items FFQ	Mediterranean	High intakes of vegetables, legumes, pasta/rice, dairy, fish/shellfish, vegetable oils, alcohol, nonsweetened nonalcoholic beverages and low intakes of processed meat.	NA
	PCA	Energy-rich	High intakes of bread/breakfast cereals, margarine, nuts, snacks/sweets and nonsweetened nonalcoholic beverages and low intakes of sweetened, nonalcoholic beverages.	Total fat, protein, Vitamin B6 and carbohydrate.
		Western	High intakes of potatoes, pasta/rice, dairy, fresh meat, processed meat, margarine and alcohol and low intakes of nuts, fish/shellfish.	NA
Colon-Ramos, U., *et al.* [63]	111-items FFQ	Healthy	High intakes of vegetables, fruits, non-fried fish and chicken, and water.	Protein and carbohydrate.
		Processed	High intakes of processed meat, fast food items, snacks, sweets, and soft drinks.	Transfats and total grains.
		Southern	High intakes of cooked cereals, peaches, corn, fried fish, beans, greens, pig’s feet, neck bones oxtails, tongue, pork.	NA
	PCA	Southern-processed	High intakes of total fat, total sugar, iron, zinc, sodium, and meats, and low intake of whole grains.	Total fat, total sugar, iron, zinc and sodium.
		Healthy-Southern	High intakes of fiber, folate, egg-meat equivalents, oils, vegetables (including dark green and orange vegetables, and tomatoes, excluding legumes and potatoes) and fruits (including fruit juice).	Fiber and folate.
		Healthy-processed	High intakes of nuts, seeds, whole grains, and dairy, as well as highly refined foods that are higher in simple sugars and fat.	Simple sugars and fat.
		Mixed	Reflects foods from all of the other patterns together.	NA
Miyake, Y., *et al.* [64]	150-items FFQ	Healthy	High intake of green and yellow vegetables, seaweed, mushrooms, white vegetables, pulses, potatoes, fish, sea products, fruit, and shellfish.	Linoleic acid, DHA, α-linolenic acid, calcium, Vitamin D, β-carotene and Vitamin E.
	Factor analysis	Western	High intake of vegetable oil, salt-containing seasonings, beef and pork, processed meat, eggs, chicken, and white vegetables.	Linoleic acid, α-linolenic acid, β-carotene and Vitamin E.
		Japanese	High intake of rice, miso soup, sea products, and fish.	DHA, calcium, Vitamins D and E.
Shaheen, S.O., *et al.* [65]	111-items FFQ	Health conscious	High intake of salad, fruit, fruit juices, rice, pasta, oat/bran based breakfast cereals, fish, pulses, cheese, non-white bread.	Protein, Mg, Fe, Zn, Na, K, niacin, fiber, thiamine, Vitamins B6, C, and folate.
		Traditional	High intake of vegetables, red meat, poultry.	Carotene, K, Vitamins B6 and C.
	PCA	Processed	High intake of meat pies, sausages, burgers, fried foods, pizza, chips, crisps, white bread, eggs, baked beans.	Monounsaturated and saturated fats, protein, carbohydrates and Na.
		Vegetarian	High intake of meat substitutes, pulses, nuts, herbal tea and low in poultry and red meat.	NA
		Confectionery	High intake of chocolate, sweets, biscuits, cakes, puddings.	Sugar.
Lange, N.E., *et al.* [35]	166-items FFQ	Prudent	High in vegetables, fruit, legumes, fish, poultry, eggs, salad dressing, and whole grains.	NA
	PCA	Western	High in red and processed meats, sugar-sweetened beverages,	NA
			French fries, high-fat dairy products, desserts, butter, and refined grains.	
Grieger, J.A., *et al.* [66]	100-items FFQ	High protein/fruit	High in fish, meat, chicken, fruit.	Long-chain *n*-3 PUFA, protein, cholesterol and Zn.
	PCA	High fat/sugar/takeaway	High in takeaway foods, crisps, refined grains.	Total fat, α-linolenic acid, SFA and Na.
		Vegetarian-type	High in vegetables, fruit, soya milk, whole grains.	Fiber, dietary folate and Vitamin A.
Steenweg-de Graaff, J., *et al.* [67]	293-items FFQ	Mediterranean	High intake of vegetables, fish & shellfish, vegetable oil, fruit, and eggs, and relatively low in processed meat.	Antioxidants and ω-3 fatty acids.
	PCA	Traditionally Dutch	High intakes of fresh and processed meat, potatoes, margarines and low intake of soy and diet products.	Saturated fat and ω-6 fatty acids.
		Confectionary	High intakes of cakes, sugar & confectionary products, tea, cereals, fruit, dairy products.	Sugar and fat.
Vujkovic, M., *et al.* [68]	178-items FFQ	Western	High intakes of organ meat, red meat, processed meat, pizza, legumes, potatoes, French fries, condiments, and mayonnaise, but low intakes of fruits.	Higher saturated fats, cholesterol monounsaturated and polyunsaturated fats and lower total proteins, carbohydrates, fiber, β-carotene, ascorbic acid, thiamin, riboflavin and pyridoxine.
	PCA	Prudent	High intakes of fish, garlic, nuts, and vegetables and a higher frequency of hot meals per day.	Higher polyunsaturated fats, fiber, Carotene, Vitamin B12 and folate.
Vujkovic, M., *et al.* [69]	200-items FFQ PCA	Mediterranean	High intakes of vegetables, fruit, legumes, vegetable oil, cereal products, alcohol and fish and low intakes of potatoes and sugar and confectionary.	Higher serum and red blood cell folate, serum vitamin B12 and lower plasma homocysteine.
de Kort, C.A., *et al.* [70]	28-items FFQ	Health conscious	High intakes of fresh fruit and vegetables, dried fruit, fresh or frozen fish, soya products, pulses, olive oil and organic food.	NA
	Cluster analysis	Mixed	High intakes of dairy products, all types of frozen or tinned products, meat, poultry and organ meats (offal).	NA
		Non-health conscious	Low intakes of yoghurt, cheese, eggs, fruit and vegetables, fish, beans and pulses, olive oil and organic food.	NA
Sotres-Alvarez, D., *et al.* [71]	58-items FFQ	Prudent	High intakes of yogurt, reduced-fat milk, whole-wheat bread, fortified cereal, fish, fruits and vegetables and low in chili peppers, avocados and beef by-product.	Almost all micronutrients except choline, iron, and Vitamins E, B6, and B12.
	LCA ^g^	Mexican	High intakes of fruits, vegetables, salsa, chili peppers, avocados, refried beans, tortillas, and chicken or beef by-products (liver, tongue) and low in reduced-fat milk, spinach and low-calorie soda.	High carbohydrates, choline, iron, Vitamins E, B6,B12 and fiber and low saturated fat, total fat, and folic acid.
		Western	High intakes of frankfurters, bacon, French fries, white bread, potato chips and regular soda and low in fruits and vegetables.	Transfat and caffeine.
		Low-calorie Western	The same as Western just 273 fewer calories than women in the Western on average.	Transfat and caffeine.
Musselman, J.R., *et al.* [72]	21-items FFQ	Western diet	High intakes of processed meats and packaged snack foods.	NA
	PCA	Fruits and vegetables	High intakes of carrots, fruits, juices, green salads, and cruciferous vegetables.	NA
		Proteins	High intakes of eggs, bacon, pork, and fried chicken.	NA
		Healthful	High intakes of skim/low-fat milk and other vegetables and low in whole milk, processed foods, and fried chicken.	NA

^a^ FFQ: food frequency questionnaire; ^b^ PCA: Principal Component Analysis; ^c^ NA: not available; ^d^ PUFA: polyunsaturated fatty acid; ^e^ SFA: saturated fatty acid; ^f^ DHA: docosahexaenoic acid; EPA: eicosapentaenooic acid; ^g^ LCA: Latent Class Analysis.

## Abbreviations

The following abbreviations are used in this manuscript:

GDM	gestational diabetes mellitus
FFQs	food frequency questionnaires
PCA	principal components analysis
LCA	Latent Class Analysis
DASH	Dietary Approaches to Stop Hypertension
aMED	alternate Mediterranean Diet
aHEI	alternate Healthy Eating Index
MUFA:SFA	the ratio of monounsaturated fatty acid to saturated fatty acid
PUFA:SFA	the ratio of polyunsaturated fatty acid to saturated fatty acid
DHA	docosahexaenoic acid
EPA	eicosapentaenooic acid
T2DM	Type 2 diabetes mellitus
HDPs	Hypertensive disorders of pregnancy
SGA	small for gestational age
NTDs	neural tube defects
GI	glycemic-index
